# Emerging two-dimensional material nanozymes for theranostic nanomedicine

**DOI:** 10.52601/bpr.2021.210011

**Published:** 2021-06-30

**Authors:** Yanling You, Zhongmin Tang, Han Lin, Jianlin Shi

**Affiliations:** 1 State Key Laboratory of High Performance Ceramics and Superfine Microstructure, Shanghai Institute of Ceramics, Chinese Academy of Sciences, Shanghai 200050, China; 2 Center for Nanomedicine, Brigham and Women’s Hospital, Harvard Medical School, Boston, Massachusetts 02115, USA

**Keywords:** Two-dimensional nanomaterials, Nanozymes, Nanomedicine

## Abstract

Nanomaterials-based artificial enzymes (nanozymes) with valuable enzyme-like catalytic properties have been booming during the past few years. Promoted by the advances in biological medicine and nanotechnology, nanozymes possess the potential to serve as an emerging agent for biosensing, immunoassays, detection and diagnosis, catalytic therapeutics, and other applications in the biomedicine field. Two-dimensional (2D) nanomaterials are of considerable interest in biomedical applications due to their ultrathin layered structure and unique physiochemical properties. Inspired by the diversified catalytic performance of 2D nanomaterials, scientists extensively have developed 2D materials as bioactive nanozymes for theranostic nanomedicine. Here, recent advances in enzyme-like 2D nanomaterials design and construction are comprehensively presented. Additionally, we exhibit that, with the synergistic effect of catalytic activities and desirable physicochemical performances, 2D nanozymes can serve as versatile platforms with extensive applications from target detection to *in vivo* theranostic. It is believed that such promising alternatives towards natural enzymes will be of vital significance in the field of nanotechnology and biomedicine.

## INTRODUCTION

Enzyme is a natural biocatalyst related to many physiological processes in living organisms known for its high efficiency and selectivity, which is one of most delicate natural products. However, the intrinsic drawbacks of natural enzymes such as fabrication and preservation difficulties, high cost, poor recyclability and laborious preparation, impede their practice applications in biomedical engineering (Lin *et al*. 2014). Inspired by biomimetic engineering, Ronald Breslow innovatively proposed the notion of artificial enzymes aiming to simulate the catalysis mechanism and process of natural enzyme by using synthetic materials (Breslow and Overman 1970). The rapid growth of researches in nanotechnology brings new opportunities to bionic enzymes owing to the nano-size effect and satisfactory properties of nanomaterials. As early as 2004, Scrimin and co-workers pioneered the term "nanozyme" and opened the door to the favorable integration of nanomaterials and enzymology (Manea *et al*. 2004).

Nanozyme is used as an umbrella term for nanomaterials or nanostructures with natural enzyme-like properties not only representing a certain kind of material, but a variety of materials combinations (Gao *et al*. 2007; Jiang *et al*. 2019; Korschelt *et al*. 2018; Liang and Yan 2019; Manea *et al*. 2004; Wei and Wang 2013). Up to now, a large number of nanozymes have been constructed to exert on-demand highly and tunable catalytic activity in various fields of chemistry, biology and medicine, by promoting the target chemical reaction (Giljohann *et al*. 2010; Huang *et al*. 2011; Kim *et al*. 2009; Lu *et al*. 2009; Sun *et al*. 2012; Wulff and Liu 2012; Xu *et al*. 2019). The interdisciplinary concept of nanocatalytic medicine (NCM) was introduced by our group to describe the catalytic therapeutic strategies that the chemical reactions can be triggered *in situ* to generate or eliminate the corresponding species, and further treating various diseases (Lin *et al*. 2018b; Yang *et al*. 2019). Notably, nanozymes completely correspond with the emerging perspectives of theranostic practices. Typically, it is well known that the overexpressed reactive oxygen species (ROS) like hydrogen peroxide (H_2_O_2_), hydroxyl radical (·OH), singlet oxygen (^1^O_2_) and superoxide anion (O_2_^−^) contribute to oxidative damage-related diseases (Vander Heiden *et al*. 2009; Zhang *et al*. 2021; Zhou *et al*. 2020). Superoxide dismutase (SOD), peroxidase (POD), and catalase (CAT) in human body are of vital importance in the regulation of ROS contents (Pisoschi and Pop 2015; Sies 2015). By mimicking the ROS scavenging capability of natural enzymes, nanozymes featuring the similar functions can clear the excessive reactive oxygen species, and thus suppressing inflammation (Liu *et al*. 2020; Qin *et al*. 2020; Wu *et al*. 2021), preventing neurological injury and aging-related diseases (Bao *et al*. 2018; Kwon *et al*. 2018; Singh *et al*. 2017), *etc*.

Exploring the structure-activity relationship towards nanomaterials and/or nanostructures has been a long-held goal for materials science. Novoselov and Geim *et al*. reported the first experimental prototype of two-dimensional (2D) single-elemental atom crystal-graphene in 2004, opening a new era of two-dimensional materials (Novoselov *et al*. 2004). Since ultrathin 2D topologic confinement, electrons in nanosheet could only enable to move freely on the non-nano scale inside planar dimension, which endows 2D materials with unique electrical characteristics and excellent physicochemical properties (Gopal *et al*. 2020; Zhang 2015). In recent, 2D nanomaterials and their potential applications in catalysis and electric industry have been widely discovered, arousing explorations of which to serve as nanozymes (Deng *et al*. 2016). In addition, owing to desirable biocompatibility and biodegradability, 2D nanomaterials hold great potential in the field of biomedicine (Cheng *et al*. 2020; Wang and Cheng 2019). Based on exceptional catalytic activity and potentials in future clinical applications, 2D nanomaterials have been reported to exhibit stimuli-responsive and multi-enzyme mimetic activities (Chen *et al*. 2018; Chong *et al*. 2016; Gao *et al*. 2020; Liu *et al*. 2021; Ren *et al*. 2019; Shan *et al*. 2019; Song *et al*. 2010; Yim *et al*. 2018). In general, the designable 2D nanomaterials represent a next-generation artificial nanozymes for the unique applications in some specific niches.

**Figure 1:**
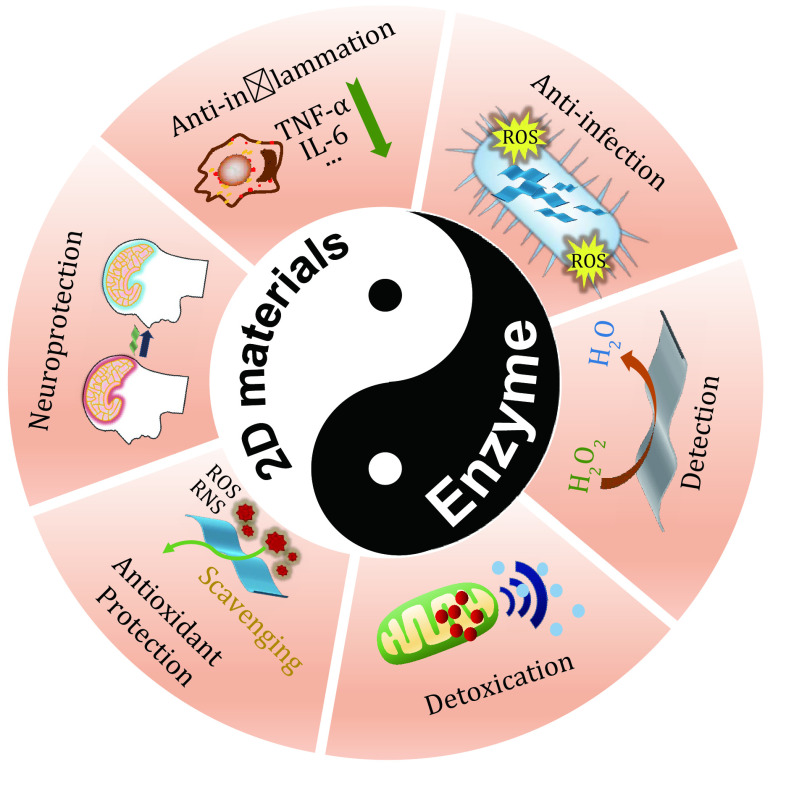
Schematic illustration of emerging two-dimensional material nanozymes for theranostic nanomedicine

Despite the rapid development of 2D nanomaterials, few studies have specifically summarized its applications as nanozymes in the field of biomedicine. To highlight the value of 2D materials in the fields of biology and medicine, this review discusses the endeavors on developing promising enzyme-like two-dimensional nanomedicine. We also introduce the latest multifunctional 2D nanozymes and present how they transform their own enzyme-like activities under the activation of the external environment to enhance the therapeutic efficacy for different diseases. Finally, we come up with future perspectives of nanozyme development with a view to improving the discipline system of "nanozymology" and promoting their practical applications (Fig. 1).

## TWO-DIMENSIONAL NANOMATERIALS AS NANOZYME FOR BIOMEDICAL APPLICATIONS

2D nanomaterials such as graphene, MXene, transition metal dichalcogenides (TMDs), black phosphorus, layered double hydroxides (LDH), and layered metal oxides, are of increasing attention for their intriguing physiochemical properties (Lin *et al*. 2018a; Zhang 2015). Surprisingly, the past decades have witnessed growing interest in developing advanced 2D nanomaterials featuring the mimicking potential of natural enzyme functions due to their high catalytic performances. In this section, we will introduce design concepts for multifunctional 2D nanomaterials of enzyme-like activity and their biomedical applications.

### Graphene and derivatives-based nanozyme

Graphene is the most representative 2D nanomaterials endowed with unique electronic, optical and other physicochemical properties, which has been widely investigated in nanomedicine and biomedical engineering (Geim and Novoselov 2007). Duo to the lack of catalytic activity, pristine graphene is inadequate to be an artificial candidate material as nanozyme (Guo and Dong 2011). Nevertheless, it provides new opportunities like a few carbon-based nanomaterials including graphene oxide (Song *et al*. 2010), fullerene (Kroto *et al*. 1985), carbon nanotubes (Cui *et al*. 2011) and carbon nanodots (Chong *et al*. 2016) have been found to possess enzyme-like activity. In specific, upon doping or modification, graphene-based nanomaterials can exhibit biocatalytic functions similar to oxidase, catalase, superoxide dismutase, *etc*. (Boutorine *et al*. 1995).

**Figure 2:**
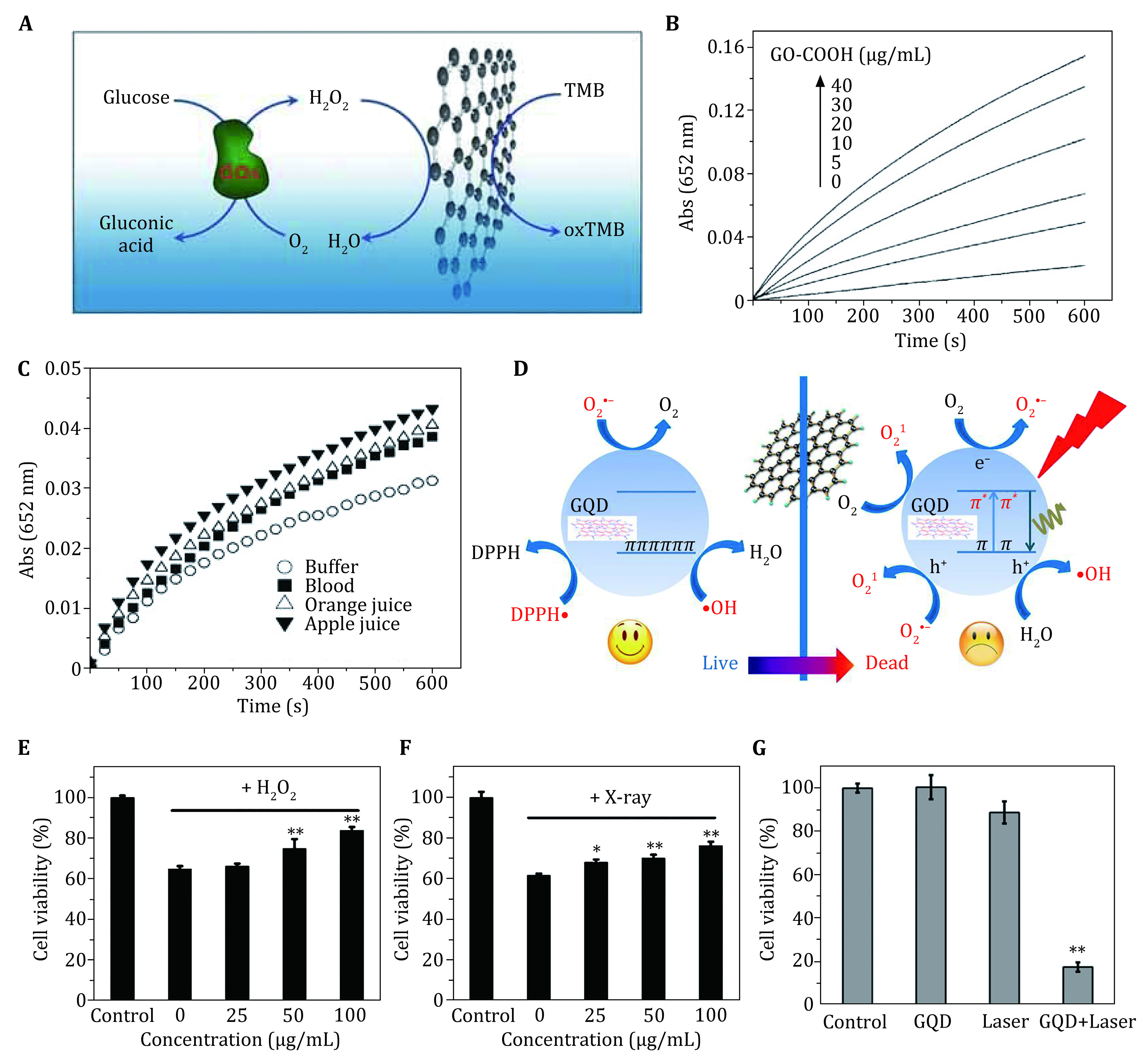
Graphene-based nanomaterials as nanozymes for detection and therapy. **A** Illustration of GO-COOH serving as a peroxidase mimic for glucose detection. **B** The absorbance change curves of the TMB oxidation reaction catalyzed by GO-COOH with varied concentrations. **C** The absorbance change curves for various samples. Reproduced with permission (Song *et al*. 2010). **D** Schematic diagram of GQDs with light-dependent ROS-regulated activity. Cell viability of HUVECs damaged by H_2_O_2_ (**E**) and X-ray (**F**) under the antioxidant protection of GQDs with varied concentrations. **G** GQDs showed completely opposite effect of ROS regulation performance under the exposure of light. Reproduced with permission (Chong *et al*. 2016)

In 2010, Song *et al.* reported that carboxyl-modified graphene oxides could catalyze the reduction of H_2_O_2_, which demonstrated intrinsic peroxidase-like activity derived from carboxyl-modified graphene oxides noted as GO-COOH (Song *et al*. 2010). To take advantage of the enzyme-like activity, they designed a colorimetric method for H_2_O_2_ and glucose detection. In the presence of H_2_O_2_ and peroxidase substrate 3,3’,5,5’-tetramethylbenzidine (TMB), GO-COOH can change TMB absorbance at 652 nm by a blue chromogenic reaction (Fig. 2A, 2B). Given that the catalytic activity of GO-COOH is relative with H_2_O_2_ concentrations, it is feasible for GO-COOH to detect H_2_O_2_. In addition, by detecting hydrogen peroxide, the product of the glucose oxidase catalyzed reaction, a simple and rapid detection of glucose was successfully developed. The detection activity of GO-COOH was regulated by acidic pH, reaction temperature, and hydrogen peroxide concentration, and the optimal conditions were of pH 4.0, 35.8 °C, and 150 mmol/L after systematic assessment. It could be found that the catalytic activity of GO-COOH towards TMB superior to that of natural enzyme horseradish peroxidase (HRP) under the optimum conditions. As the Fig. 2C shows, the GO-COOH nanozyme presents highly selective and sensitive detection performance of glucose in buffer solution, dilute blood and fruit juice. H_2_O_2_ detection is of great significance in the fields of biology, medicine, environmental protection, chemical industry and food engineering. Inspired by the participation of H_2_O_2_ in the catalytic reaction of peroxidase, the concentration of H_2_O_2_ can be detected selectively by the color reaction of peroxidase-like nanozymes with certain colorimetric substrates. More importantly, the designing concept is not limited to H_2_O_2_ peroxide detection. Such study provides a new insight into applying nanozymes in targeting detection and diagnosis.

In order to further promote the applications of graphene, scientists have developed various methods to convert 2D graphene to 0D graphene quantum dots (GQDs). As a typical structural derivative of graphene, quantum dots are of strong photoluminescence property which is essential to several applications like bioimaging or photovoltaics. Additionally, the photoluminescent GQDs could react with either electron acceptor or donor, and subsequently be quenched, which may serve as an emerging free radical scavenging nanoagent. For instance, Chong and co-workers discovered that graphene quantum dots have ROS-regulation function that GQDs could perform superoxide dismutase-like activity to downgrade ROS levels, while generating ROS to produce phototoxicity upon exposure to blue light (Fig. 2D) (Chong *et al*. 2016). This exceptional Janus-like ROS-regulated performance simultaneously enables GQDs with oxidative stress relieving capacity and antibacterial or anticancer efficacy. Based on desired antioxidant activity, they explored the effectiveness of GQDs protecting cells from oxidative damage *in vitro* and *in vivo*. The cell experiments indicated that the GQDs remarkably improved the survival rate of H_2_O_2_ and X-ray damage cells (Fig. 2E, 2F). Furthermore, they found that under the exposure of light, GQDs showed completely opposite effect of ROS regulation performance that GQDs elicit phototoxicity under the 405 nm laser irradiation by generating ROS and further inducing cell apoptosis (Fig. 2G). What’s more, GQDs could accelerate the inactivation of anti-oxidase in body. This research developed a light-activatable nanozyme and demonstrated the artificial nanomaterials of diverse bio-functions through conditional activation. Since most 2D nanomaterials are known to feature photoelectric characteristics, it is of great significance to develop stimuli-responsive smart nanomedicine based on diverse 2D nanomaterials.

### Two-dimensional (2D) transition metal oxides (TMOs) -based nanozymes

During the past few years, transition metal oxides (TMOs) have received great attention and spurred a range of research efforts. 2D TMOs nanosheets with specific mixed valence, desirable conductivity property, abundant redox combinations, and thermal stability have attracted current interest in various applications such as photoelectric catalysis, sensors, and electrochemical energy storage (Haque *et al*. 2017; Lee *et al*. 2018).

Vanadium is a transition metal. It is widely believed that vanadium pentoxide (V_2_O_5_) is the toxic compound, which is an oxidant and a common component in PM_2.5_ (Dill *et al*. 2004). Interestingly, a great deal of novel properties is emerging as materials are being decreased to nanoscale. V_2_O_5_ materials at nanoscale show completely opposite characteristics. For example, a biocompatible nanozyme based on V_2_O_5_ nanowires with remarkable glutathione peroxidase (GPX)-like activity was developed for performing ROS scavenging and serving as cytoprotectant against harmful oxidative damage (Vernekar *et al*. 2014).

**Figure 3:**
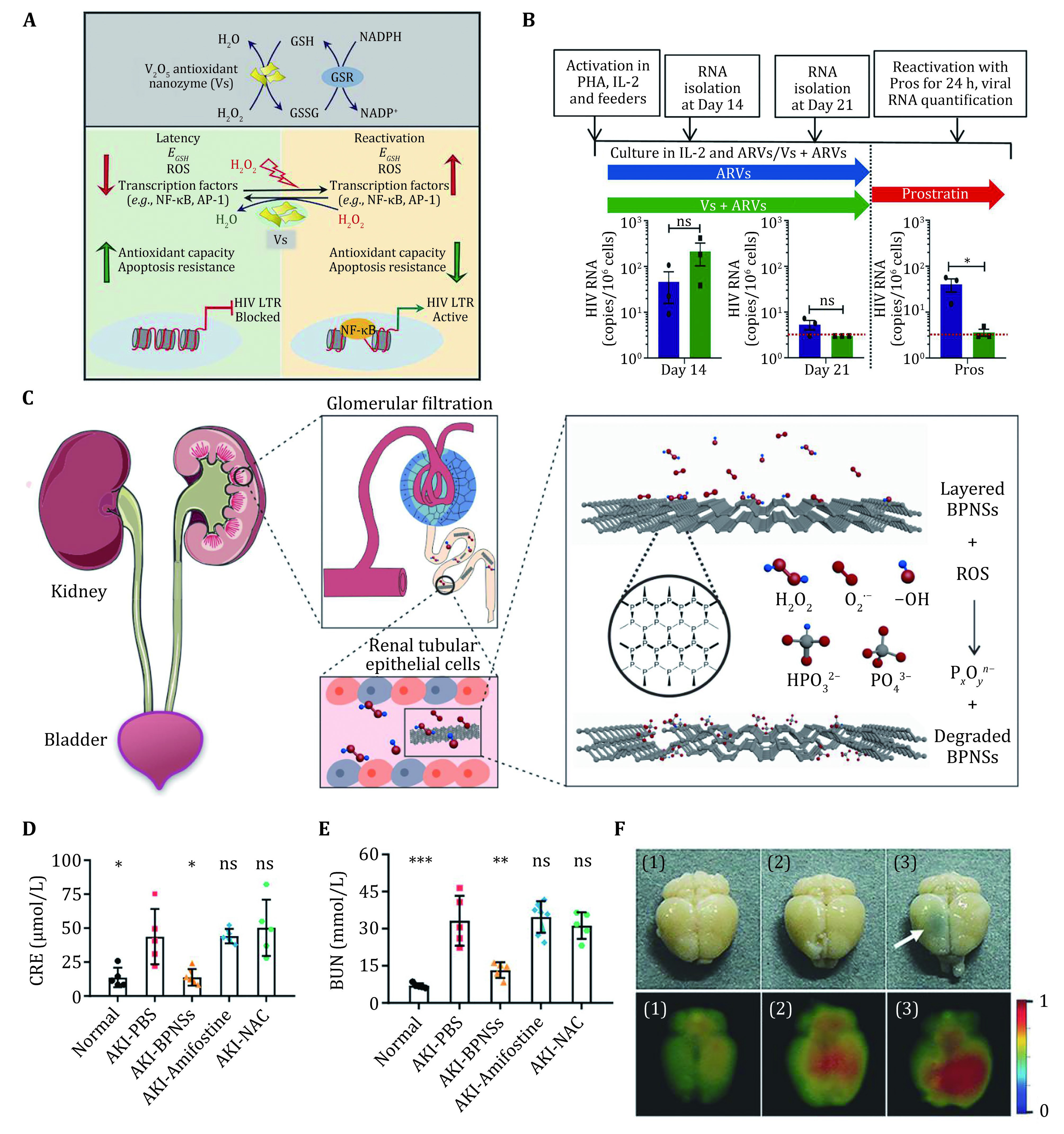
Antioxidant nanozyme for various diseases treatment. **A** Schematic illustration of V_2_O_5_-based nanozymes for anti-HIV-1 infection. **B **The activation of viral transcription in CD4^+^ T cells from three HIV-1-infected patients in the treatment of V_2_O_5_ nanosheets. HIV transcripts measured by RT-PCR. Reproduced with permission (Singh *et al*. 2021). **C** Experimental illustration of black phosphorus nanosheets for acute kidney injury therapy. **D**, **E** Blood analysis of mice from different groups. Reproduced with permission (Hou *et al*. 2020). **F** Photographs and the NIR fluorescence imaging of brains after various treatments (1. Cy5-PEG-BP; 2. Cy5-PEG + NIR; 3. Cy5-PEG-BP + NIR). Reproduced with permission (Chen *et al*. 2018)

Recently, a few investigations have reported that reactive oxygen species (ROS) levels are associated with the replication of human immunodeficiency virus (HIV-1) after infection. Cellular antioxidant machinery has been demonstrated to contribute to the acceleration of HIV-1 reactivation during HIV-1 latency (Diaz *et al*. 2019; Shytaj *et al*. 2020). In order to neutralize ROS in HIV-1 infected cells and inhibit viral reactivation, Singh *et al*. used V_2_O_5_ nanosheets (Vs) as glutathione peroxidase-like nanozyme, which is the pioneering therapeutic strategy against HIV infection (Fig. 3A) (Singh *et al*. 2021). In 2018, Ghosh *et al*. found that the enzyme‐mimetic activity of V_2_O_5_ was dependent on the reactivity of V=O bond and exposed facets on the surface rather than the morphology (Ghosh *et al*. 2018). A comparison of activities and toxicities of V_2_O_5_ in three different morphologies, *i.e*., nanowires, nanosheets, and ultrathin nanosheets was conducted by Singh and co-workers. As a result, they found that ultrathin V_2_O_5_ nanosheets were selective toward H_2_O_2_ with high efficiency and the most secure, which also confirmed the unique superiority of 2D nanomaterials in biomedicine applications. As shown in Fig. 3B, the viral RNA levels from Vs treatment groups were obviously lower than the groups of ARVs (antiretrovirals) alone at Day 21. Meanwhile, V_2_O_5_ nanosheets could also suppress viral reactivation stimulated by prostratin and bolster the antiviral capacity of immune cells. It is a great discovery that oxidative stress which changes redox signaling promotes the reactivation and multiplication of HIV-1, revealing the relationship between redox metabolism and disease outcomes. These advances together offer a novel therapeutic potential for the HIV-1 adjuvant therapy of diverse antioxidants especially nanozyme. The satisfactory glutathione peroxidase (GPX)-like activity of V_2_O_5_ nanosheets may encourage new applications to treat other critical illness with redox imbalance.

### Black phosphorus nanosheet-based nanozymes

Black phosphorus nanosheet (BP NS) marks an omnipotent, rising-star nanomaterial with unique properties. Remarkably, BP is also a promising candidate in the multidisciplinary biomedical field due to its good biocompatibility and excellent biodegradation. In the past few years, BP exhibited considerable photothermal therapy (PTT) and photodynamic therapy (PDT) efficacies (Wang *et al*. 2015). In recent, researchers have been focusing on the enzyme-like activity of BP, owing to its prior chemical reactivity with ROS. This finding introduces BP as a new species of nanozyme for anti-oxidant related diseases therapeutics.

As stated, acute kidney injury (AKI) as a severe illness leading to loss of renal function is largely caused by drugs administration in chemotherapy. In the process of the renal excretion of chemotherapeutic drugs, massive ROS generate and damage renal cells. In order to relieve AKI and protect the renal function, Hou *et al*. prepared a black phosphorus-based anti-oxidative nanoagent for kidney targeting and ROS neutralizing (Fig. 3C) (Hou *et al*. 2020). It has been reported that flake-like DNA frameworks have ability to kidney targeting drug delivery (Jiang *et al*. 2018). The same geometrical framework endows BP NSs with passive targeting to kidney, and the biocompatible nanosheets can degrade into nontoxic phosphorus oxides and/or phosphates. Most importantly, BP NSs display fascinating performance without any sophisticated modifications which facilitates their ultimate clinical translation potential. The blood biochemical indexes of renal function including serum creatinine (CRE) and blood urea nitrogen (BUN) elucidated that the BP NSs are an effective therapeutic antioxidant as a SOD mimic for AKI treatment (Fig. 3D, 3E). Notably, the outstanding biocompatibility and biodegradability of BP NSs promote the further translational research on such a promising 2D nanozyme for AKI treatments. Furthermore, the *in vivo* imaging diagnosis shows great value in real-time monitoring for AKI (Wang *et al*. 2021). 2D nanomaterials-based contrast agents are also expected to provide multiple applications in diagnostic-imaging fields, which will allow innovative theranostics in AKI.

Growing evidence suggests that the elevation of transition-metal ions is the main cause of many neurodegenerative diseases (Barnham and Bush 2014; Jun and Saxena 2007). Except for ROS scavenging, BP NSs may also aid in the regulation of metal ions such as Cu^2+^ for neurodegenerative disorder treatment. For example, Chen *et al*. used BP NSs as a neurodegenerative disorder therapeutic agent which can cross the blood–brain barrier (BBB) and capture excess redox-active Cu^2+^ (Chen *et al*. 2018). Notably, their work exhibited a synergistic therapeutic strategy that BP nanosheets could effectively enhance the blood brain barrier penetration due to local hyperthermia taking advantage of the high photothermal-conversion efficiency (Fig. 3F). Subsequently, BP nanosheets as neuronal cells protector enables to downgrade the cellular Cu^2+^ level and eliminate ROS. Thus, it is believed that black phosphorus nanosheet will be applied as a SOD-like nanozyme which may perform multifunctional nanodrugs rather than just conduct ROS scavenging.

### 2D transition metal chalcogenides (TMCs) nanosheet-based nanozyme

2D transition metal chalcogenides (TMCs) containing transition metal atoms and sulfur atoms are of great interest in different fields especially energy storage, catalysis, electroluminescent devices, bioimaging and nanomedicine (Manzeli *et al*. 2017). It is novel but highly attractive that 2D TMCs nanomaterials possess the ability to scavenge ROS and/or reactive nitrogen species (RNS) via reducing free radicals similar to superoxide dismutase and nitrate reductase (NR). The intrinsic connection between 2D nanomaterials and natural enzymes has opened the door for providing new strategy of free radical scavenging and ROS or RNS-related disease therapeutic modalities.

**Figure 4:**
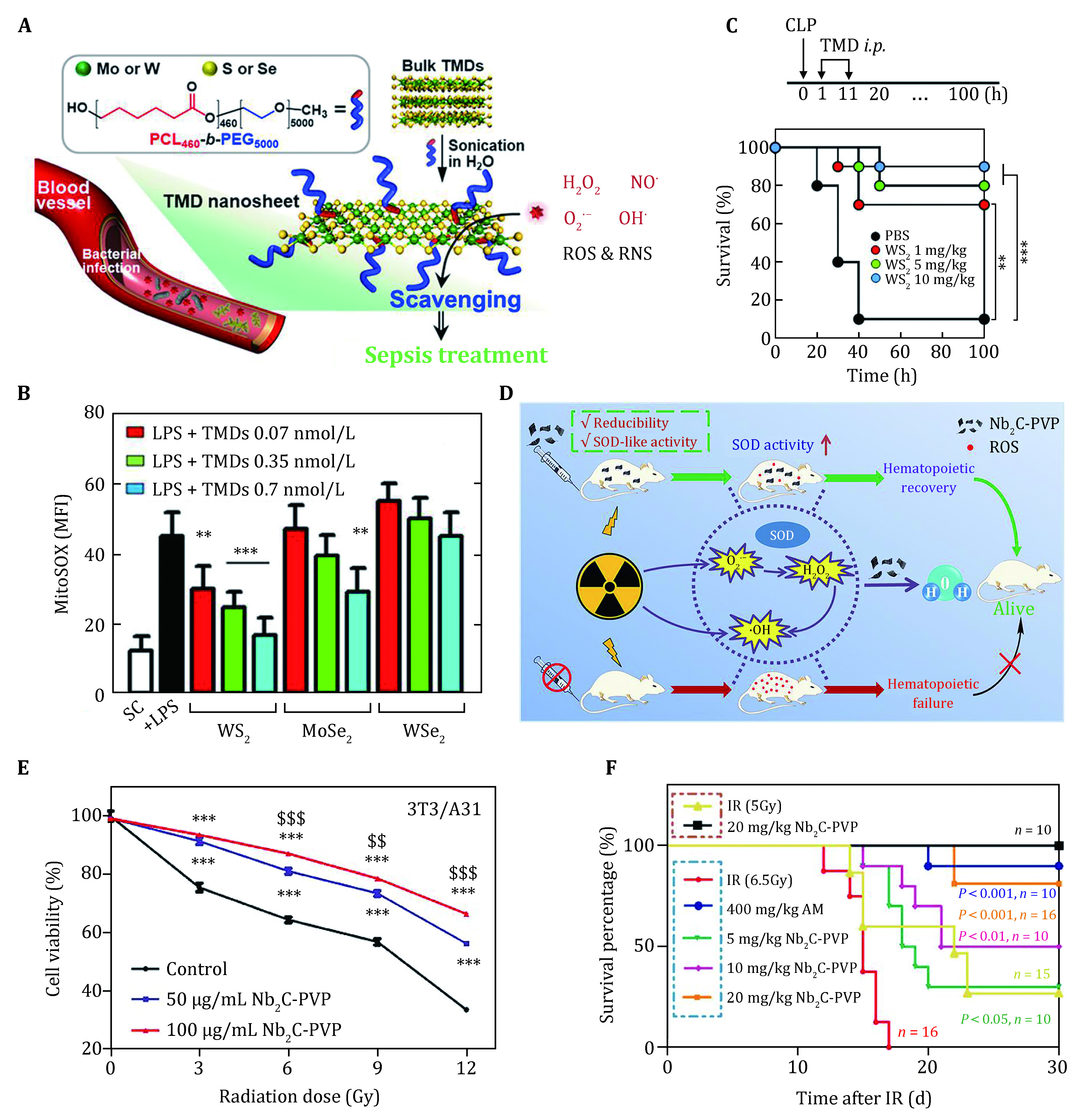
TMCs nanosheets and MXenes scavenging activity. **A** Schematic diagram for the functionalization of TMD nanosheets and their ROS and RNS scavenging for sepsis treatment. **B** ROS and RNS levels in LPS-induced inflammatory BMDMs incubated with different TMD nanosheets. **C** 100 h survival rate of septic mice with treatment of 0, 1, 5, and 10 mg/kg of WS_2_. Reproduced with permission (Yim *et al*. 2020). **D** Schematic illustration of Nb_2_C-PVP with SOD-like activity to reduce IR-induced damage. **E** Cell viability of 3T3/A31 cells after treatment with Nb_2_C-PVP suspension at varied concentrations. **F** Survival percentage of BALB/C mice under the radiation of sub-lethal TBI and lethal TBI with different pretreatments. Reproduced with permission (Ren *et al*. 2019)

As a typical paradigm, a biocompatible enzyme-like nanomedicine based on 2D TMCs nanosheets with effective antioxidative activity was developed for sepsis treatment (Fig. 4A) (Yim *et al*. 2020). In particular, the functionalized TMCs could reduce cellular ROS and RNS without disrupting ROS-producing enzymes. The results showed that, upon stimulation with lipopolysaccharide (LPS)/toll-like receptor 4 (TLR4), the inflammatory bone marrow-derived macrophages (BMDMs) generated considerable mitochondrial ROS, and WS_2_ nanosheet exhibited superior ROS scavenging ability in contrast to that of other types of TMCs (Fig. 4B). As shown in Fig. 4C, the survival fractions of bacteremia mouse model dramatically declined, while that of mice with WS_2_ nanosheets treatment is up to 90%. Moreover, ultrathin thickness of the 2D nanosheet endows them easy excretion out of the body. Such excellent therapeutic effect and biocompatibility of 2D TMC nanoagent facilitate their versatile biomedical applications, especially in the treatment of oxidative stress-related inflammatory diseases.

### MXene nanosheet-based nanozyme

MXenes are a new class of 2D layered nanomaterials, where M stands for the transition metal, and X is carbon and/or nitrogen. In recent years, MXenes with appealing properties such as photothermal conversion, rich surface functional groups, good hydrophilicity and other biological effects, have made great progresses in the field of biomedical applications (Lin *et al*. 2018a). A recent report showed that 2D MXenes could react with free radicals, for which it can exert the effects of ROS scavenging like superoxide dismutase and catalase (CAT) (Feng *et al*. 2021).

To explore new biologic roles of MXenes, Ren *et al*. developed 2D Nb_2_C MXene as a radioprotectant for avoiding ionizing radiation (IR)-induced injury (Fig. 4D) (Ren *et al*. 2019). It is an unneglectable problem that ionizing radiation (IR) lead to serious health hazards such as acute/chronic radiation syndrome and leukemia. However, suffering from disappointing protective efficiencies and the severe side effects, current radioprotectors do very little to avert IR-induced multiple tissue injuries. MXenes are of great potential in prevention ionizing radiation with higher efficiency and biocompatibility. The CCK-8 assay exhibited that the survival rate of 3T3/A31 cells with the incubation of Nb_2_C-PVP was much higher than that without protection (Fig. 4E). They found that the pretreatment with Nb_2_C-PVP yielded a 100% survival rate of mice after γ-irradiation which evidencing a highly efficient nanozyme for attenuating IR-induced injury *in vivo* (Fig. 4F). The protective effect is much better than that of other nano-radioprotector like cysteine-protected MoS_2_ nanodots of 79% (Zhang *et al*. 2016), and Fe@C nanoshields of 90% (Wang *et al*. 2018). Furthermore, they proposed the radical-clearance mechanism of Nb_2_C via DFT calculations. It could be found that [Nb_2_C] site is the favorable adsorption site for ·OH. After continuous attack of ·OH, the hydroxyl groups on the surface can be dehydrated, forming oxygenic nanosheet of NbO_x_ species. This study paves a new avenue for developing MXenes in the field of mimicking natural enzymes for ROS quenching.

### 2D metal-organic framework (MOF) nanosheet-based nanozyme

As a novel layered nanomaterial, 2D metal-organic framework (MOF) nanosheets come into spotlight owing to the abundant active sites on their surface or pore structure in contrast to traditional 3D MOF materials. Metalloporphyrins have already proven high similarity of their microstructure together withnatural enzymes (Feng *et al*. 2012). Therefore, metalloporphyrin frameworks have been designed and established as biological oxidase-mimicking nanozymes for the catalytic activation of molecular oxygen.

**Figure 5:**
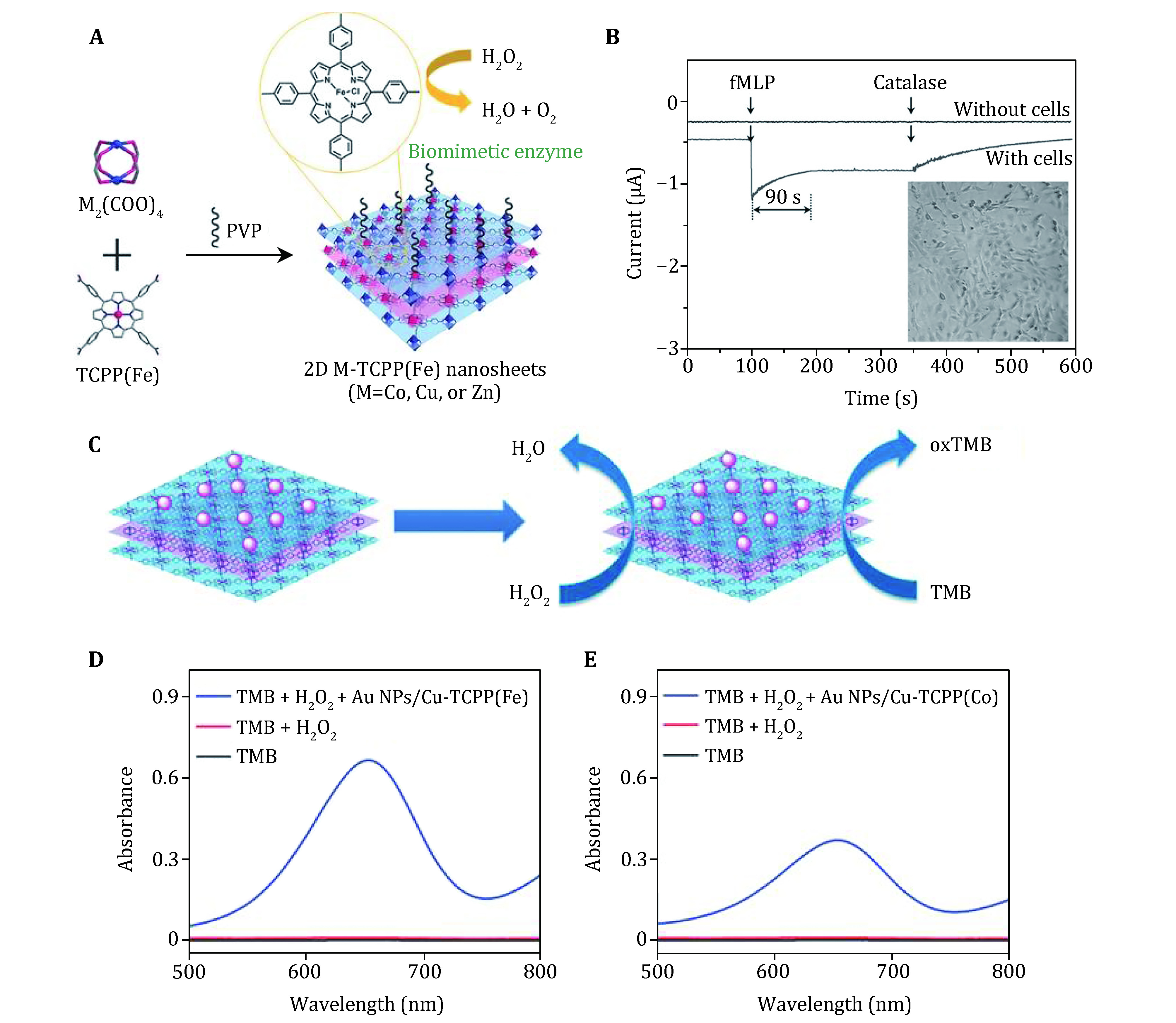
2D MOF nanosheets for biosensing application. **A** Schematic illustration of the fabrication of 2D M-TCPP (Fe) nanosheets as biomimetic enzyme. **B** The real-time monitoring of H_2_O_2_ secreted by MDA-MB-231 cells. Reproduced with permission (Wang *et al*. 2016). **C** Schematic of AuNPs/Cu-TCPP(Fe) hybrid nanosheets catalyzing the cascade reactions. **D**, **E** The UV–Vis absorption spectra of different solutions. Reproduced with permission (Huang *et al*. 2017)

In 2016, Wang *et al*. synthesized ultrathin bimetallic 2D Co-TCPP(Fe) ((Fe(III) tetra(4-carboxyphenyl) porphins chloride) nanosheets as a promising biosensor for H_2_O_2_ detection (Fig. 5A) (Wang *et al*. 2016). It is common knowledge that the iron-porphyrin derivative exhibits similar effects of hemoglobin. Inspired of the hemeprotein-like activity, researchers designed an electrochemical detection built on Co-TCPP(Fe). As expected, the glassy carbon electrode (GC)/(Co-TCPP(Fe))*_n_* (*n* = 1–6) electrodes exhibit noticeable reduction peak elucidating the reduction of H_2_O_2_, suggesting the hemeprotein-mimic activity. These results indicated that the catalytic activity of electrode exhibit a strong dependence of H_2_O_2_ concentration, showing linear relationships between the H_2_O_2_ concentration and current response with H_2_O_2_ concentration ranging from 0.4 × 10^−6^ mol/L to 50 × 10^−6^ mol/L. Furthermore, this Co-TCPP(Fe)-enabled nanozyme biosensor can effectively offer the real-time detection of H_2_O_2_ secreted from living cells (Fig. 5B).

Likewise, their group successfully mimicked enzyme triggering cascade reactions by synergistic cooperation of Au nanoparticles (NPs) and 2D Cu-TCPP(Fe) nanosheets (Huang *et al*. 2017). In this work, they elaborately prepared Au NPs/Cu-TCPP(Fe) hybrid nanosheets in which AuNPs function as oxidase-like nanozyme, while Cu-TCPP(Fe) is employed as peroxidase-mimic nanozyme (Fig. 5C). They constructed an artificial enzymatic cascade reaction system. Firstly, the hybrid nanozyme can first catalyze glucose to generate hydrogen peroxide. Secondly, the product H_2_O_2_ can oxidize TMB to produce a blue-color reaction in the presence of peroxidase-like nanozyme. As depicted in Fig. 5D and 5E, a stronger light absorption at 652 nm demonstrated that the coupled nanozyme system is capable of initiating the TMB oxidation, and the results proved the occurrence of biomimetic cascade catalytic reaction. This study verified that the artificial enzymatic cascade reactions are of great application potential by integrating diverse nanomaterials with enzyme-like properties.

### Other 2D families nanosheet-based nanozyme

**Figure 6:**
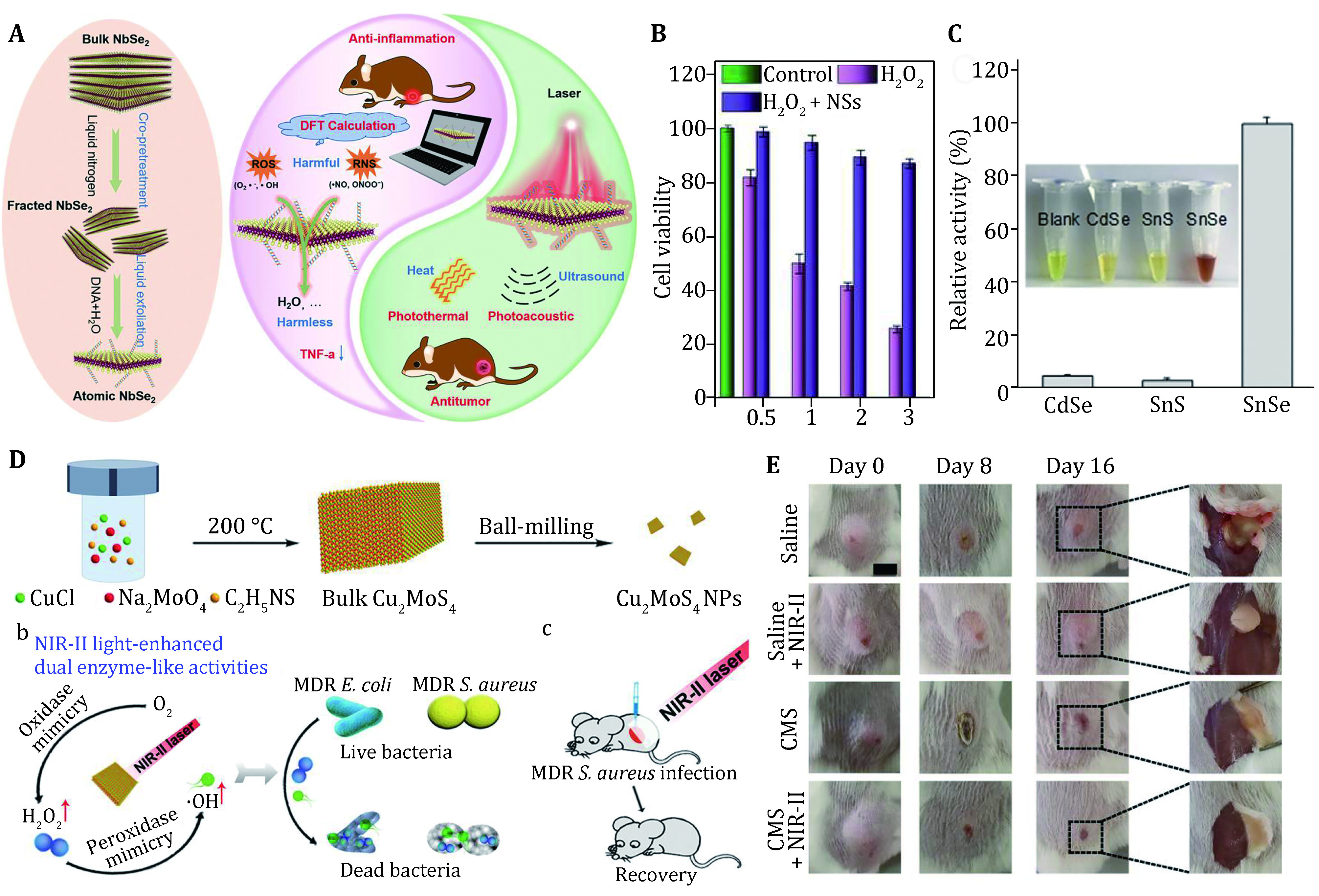
2D materials mimic enzyme-catalyzed reactions. **A** Schematic illustration of NbSe_2_ nanosheets for anti-inflammation and light-induced anti-cancer therapy. **B** Cell viability of HUVECs under H_2_O_2_ environment. Reproduced with permission (Miao *et al*. 2020). **C** The evaluation of dehydrogenase-like activity of different nanosheets, *i.e*., CdSe, SnS, SnSe. Reproduced with permission (Gao *et al*. 2020). **D** Schematic of NIR-II responsive Cu_2_MoS_4_ nanosheets of dual enzyme-like activities. **E** The photographs of MDR *S. aureus*-infected mice after different treatments. Reproduced with permission (Shan *et al*. 2020)

In addition to the 2D nanomaterials with enzyme-like characteristics mentioned above, there are numerous 2D materials used as nanozymes for catalytic medicine-related treatments. Niobium dieseline (NbSe_2_) is a kind of 2D transition metal selenides (TMSs) which holds great promise for electrical devices by virtue of its excellent superconductivity. In 2020, Miao *et al*. pioneered the application of DNA-capped NbSe_2_ as nanozyme in reactive oxygen and nitrogen species (RONS) elimination (Fig. 6A) (Miao *et al*. 2020). As discussed above, RONS signaling molecules are inflammatory signaling molecule which can induce the secretion of inflammatory cytokines and then exacerbate inflammation. It is highly expected that NbSe_2_ nanosheets possess the reaction activity with polar molecules, suggesting that the potential of nanosheets as anti-inflammatory nanodrug. As shown in Fig. 6B, NbSe_2_ exhibits evident efficacy in antioxidative protection, while H_2_O_2_ leads to massive cell apoptosis. Furthermore, these NbSe_2_ nanosheets significantly down-regulated the inflammatory cytokines level in rear thigh inflammation mice, suggesting the excellent anti-inflammatory activity *in vivo*. Moreover, under the 808 nm laser irradiation, the NbSe_2_ nanosheets exhibited favorable photothermal tumor eradication and PA imaging contrast enhancement function.

Dehydrogenase (*e.g*., malate dehydrogenase, NADH dehydrogenase and succinic dehydrogenase) is a class of vital enzymes that mediate oxidation-reduction biochemical reactions and play a key role in tricarboxylic acid (TCA) cycle (Spinelli *et al*. 2017). With the in-depth studies of disease mechanism, dehydrogenase has been found to associated with immune response and the progression of a great variety of diseases. For example, dehydrogenase may generate excessive ROS and cause oxidative stress in mitochondria, which triggers the inflammatory reaction. A representative study by Gao *et al*. firstly presented that SnSe nanosheets feature dehydrogenase-like activity, proving a novel type of nanozyme for efficient bio-catalysis (Gao *et al*. 2020). Colorimetry-based detection was employed to investigate the catalytic activities of SnSe nanosheets. Once receiving transferred hydrogen, the corresponding assay kits will display dramatic color reaction (Fig. 6C). As a result, SnSe exhibited strong ability of dehydrogenation in comparison with other similar 2D TMSs, indicating that the Sn elements exist the high catalytic effect in combination with selenium components. Given that SnSe nanosheets displayed enhanced and universal catalytic activity (>75%) in diverse reaction without the assistance of co-enzymes, it is highly expected that SnSe nanozyme will be further exploited for biomedical practices.

In the past decade, various nanomaterials as antibacterial agents have attracted considerable interests, which promote the exploration of potential antibacterial strategies that exploit different properties of materials (Xin *et al*. 2019). Catalytic medicine based on nanozymes paves a new way for bacterial infections. Aiming at multidrug-resistant (MDR) bacteria, Shan *et al*. proposed a second near-infrared window (NIR-II) light responsive nanozyme to combat multiple drug resistance (MDR) *Escherichia coli* (*E. coli*) and MDR *Staphylococcus aureus* (*S. aureus*) using Cu_2_MoS_4_ nanoplates (CMS NPs) as an oxidase- and peroxidase-like catalyst whose activity can be modulated by NIR-II laser irradiation (Fig. 6D) (Shan *et al*. 2020). Cu_2_MoS_4_ (CMS) is a neotype ternary layered TMC featuring high photothermal conversion efficiency (37.8%) and the catalytic ROS generation ability. More importantly, they found that the photothermal effect for hyperthermia facilitates ROS production to kill bacteria. Finally, a significant abscess and severe ulceration were identified in control groups showing the infection gradually aggravated without CMS NPs treatment (Fig. 6E).

## CONCLUSIONS AND PERSPECTIVES

2D nanomaterials show great promise as substitutes of natural enzymes due to their intrinsic catalytic activity. Excitedly, the superior catalytic properties of nanozyme fit neatly to the catalytic strategy for many kinds of diseases treatment. This review briefly overviews recent advances in the design and development of nanozyme based on 2D materials focusing particularly on their biological applications including detection, anti-inflammation and anti-bacteria (Table 1).

**Table 1:** Applications of two-dimensional materials-based nanozyme in biomedicine

Material	Enzyme-like activity	Application	Reference
GO-COOH, GQDs	Oxidase, catalase, superoxide dismutase	Glucose detection, antioxidant protection	Song *et al*. 2010, Chong *et al*. 2016
V_2_O_5_ nanosheets	Glutathione peroxidase	Anti-HIV-1 infection	Singh *et al*. 2021
Black phosphorus nanosheets	Superoxide dismutase	Acute kidney injury therapy, neurodegenerative disorder therapy	Hou *et al*. 2020, Chen *et al*. 2018
2D transition metal chalcogenides	Superoxide dismutase, nitrate reductase	Sepsis treatment	Yim *et al*. 2020
Nb_2_C (MXene)	Superoxide dismutase	Radioprotectant	Ren *et al*. 2019
2D MOF	Catalase, peroxidase	H_2_O_2_ detection, glucose detection	Wang *et al*. 2016, Huang *et al*. 2017
NbSe_2_	Superoxide dismutase, nitrate reductase	Anti-inflammation	Miao *et al*. 2020
SnSe	Dehydrogenase	Dehydrogenation catalysis	Gao *et al*. 2020
Cu_2_MoS_4_	Oxidase, peroxidase	Antibacterial treatment	Shan *et al*. 2020

During the past few years, there have been hundreds of literatures focused on nanozymes and a number of nanomaterials have been developed as nanozyme. Nevertheless, the area of 2D nanomaterials as nanozymes is still in its infancy. Compared to other non-2D nanozymes, 2D nanomaterials have their fascinating advantages thanking to the unique layered structure.

(1) The high specific surface areas enable the 2D nanomaterials with a high density of surface active sites, which is beneficial to enzyme-like catalytic reactions.

(2) Traditional inorganic enzyme-like nanomaterials have the unavoidable drawbacks such as poor chemical/thermal stabilities, single functionality and poor degradability. Excitedly, 2D nanomaterials possess desirable characteristics like facile modification and functionalization, tunable morphology/structure, plasticity, multivalent interactions, satisfactory biodegradation, and relatively high physiological stability, which endow 2D nanomaterials with great potential for further clinical applications.

(3) The unique inherent optoelectronic properties of 2D nanomaterials facilitate the implementation of stimuli-responsive multifunctional nanodrugs.

As a burgeoning field, the exact concept of nanozyme has not be put forward and it is urgent to establish evaluation standards for enzyme-like catalytic activity of a variety of nanozymes. Meanwhile, the catalytic mechanisms have to be comprehensively explored to allow more designs over the enzyme-like catalyst. Although extensive researches have demonstrated that SOD-like nanozymes are effective in treating ROS-related diseases, the mechanisms of treatment always elude explanation. It is urgent to thoroughly investigate the biological phenomena basing on molecular biology before applying nanozyme-drugs into human body. Moreover, when it comes to nanomedicine *in vivo* applications, biocompatibility and biosafety are the eternal theme. Comprehensive and long-term bio-safety assessment are indispensable for 2D nanozyme in future clinical applications. Enzyme is a huge family, and thousands of enzymes have been found in the human body. In addition to several enzymes that have been already studied, the development of nanozymes mimicking more types of natural enzymes is needed, which may be a new opportunity to explore and treat intractable diseases. For 2D nanomaterials, we can take advantage of their other unique physicochemical properties to catalyze reactions more than just redox type reactions. It is believed that conditional-activated smart nanozyme that can convert its functions depending upon different diseases will be a hot topic in nanomedicine field. Given that, we believe that 2D nanomaterials will be a huge part of natural enzyme alternatives offering new opportunities for novel nanozymes with a board of applications in detection, diagnostics and catalytic therapy.

## Conflict of interest

Yanling You, Zhongmin Tang, Han Lin and Jianlin Shi declare that they have no conflict of interest.
